# Toward improved *in vitro* models of human cancer

**DOI:** 10.1063/5.0026857

**Published:** 2021-01-21

**Authors:** Jose M. Ayuso, Keon-Young Park, María Virumbrales-Muñoz, David J. Beebe

**Affiliations:** 1 Department of Biomedical Engineering, University of Wisconsin, Madison, Wisconsin 53705, USA; 2 The University of Wisconsin Carbone Cancer Center, University of Wisconsin, Madison, Wisconsin 53706, USA; 3 Department of Surgery, University of California San Francisco, San Francisco, California 94143, USA; 4 Department of Pathology and Laboratory Medicine, University of Wisconsin School of Medicine and Public Health, Madison, Wisconsin 53705, USA

## Abstract

Cancer is a leading cause of death across the world and continues to increase in incidence. Despite years of research, multiple tumors (e.g., glioblastoma, pancreatic cancer) still have limited treatment options in the clinic. Additionally, the attrition rate and cost of drug development have continued to increase. This trend is partly explained by the poor predictive power of traditional *in vitro* tools and animal models. Moreover, multiple studies have highlighted that cell culture in traditional Petri dishes commonly fail to predict drug sensitivity. Conversely, animal models present differences in tumor biology compared with human pathologies, explaining why promising therapies tested in animal models often fail when tested in humans. The surging complexity of patient management with the advent of cancer vaccines, immunotherapy, and precision medicine demands more robust and patient-specific tools to better inform our understanding and treatment of human cancer. Advances in stem cell biology, microfluidics, and cell culture have led to the development of sophisticated bioengineered microscale organotypic models (BMOMs) that could fill this gap. In this Perspective, we discuss the advantages and limitations of patient-specific BMOMs to improve our understanding of cancer and how these tools can help to confer insight into predicting patient response to therapy.

## ADVANTAGES OF BIOENGINEERED MICROFLUIDIC ORGANOTYPIC MODELS (BMOMs)

**FIG. 1.**
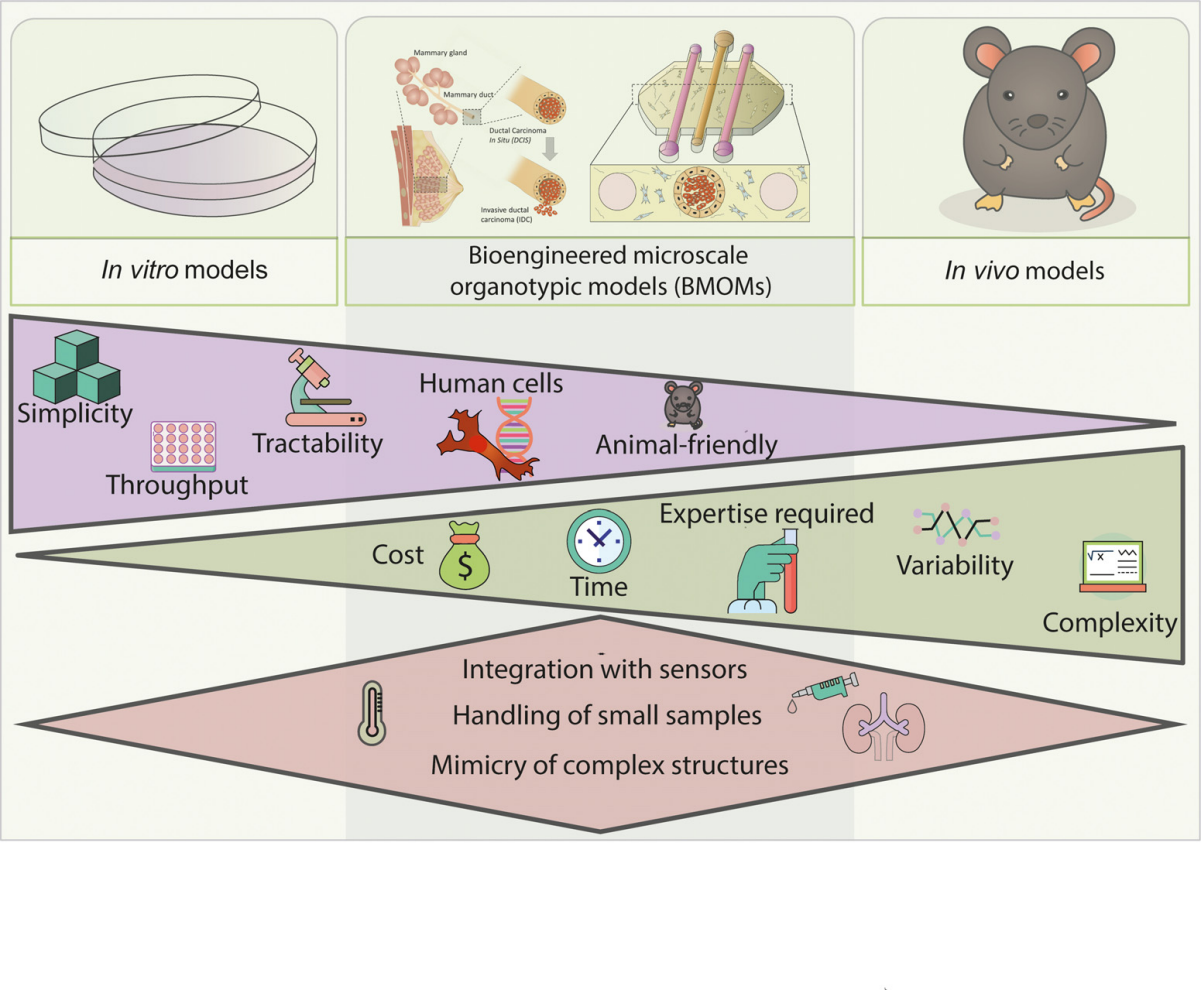
Schematic representation of a comparison among bioengineered microscale organotypic models (BMOMs), *in vitro* and *in vivo* models. BMOMs are a balanced middle ground between the advantages of *in vitro* models (purple) and the advantages of *in vivo* models (green). Further, the leverage of microscale technologies comes with advantages over both *in vitro* and *in vivo* models (red).

BMOMs are commonly defined as microscale *in vitro* platforms that rely on the use of three dimensional (3D) environments (e.g., multicellular spheroids), 3D matrices (e.g., collagen), and/or the culture of one or multiple cell types (e.g., tumor cells) to mimic specific features of *in vivo* organ physiology.^1,2^ BMOMs are based on the use of engineered microfluidic devices (i.e., with a volume capacity in the *μ*l range), which may include microchambers connected by microchannels and other microscale features. BMOMs enable researchers to control cell organization and compartmentalization as well as the flow of nutrient and waste products,^3^ which helps us to illustrate their advantage over traditional 2D platforms. In this article, we focus on the advantages derived from leveraging microscale physics, monitoring capabilities, and BMOM's bottom-up approach to modeling with all-human materials. An overview of the advantages and disadvantages of microfluidic models compared to *in vitro* and *in vivo* models is illustrated in Fig. 1 and is discussed more in-depth in this section.

### BMOMs leverage physics at the microscale

Microfluidics is defined as the manipulation of fluid volumes at the submillimeter scale. At the microscale level, the effects of surface tension and capillarity dominate gravity and inertia, which makes fluid behavior highly predictable.^4–6^ The predictability of these systems can be leveraged to control microenvironmental conditions, generate biochemical gradients, or separate components from a complex sample (e.g., multiple cell types such as tumors and blood cells).^3^

An often-mentioned advantage of BMOMs is their small sample requirement, which enables work with limited samples (e.g., patient-derived biopsies, exosomes, circulating tumor cells from the blood).^7,8^ BMOMs typically require a working volume in the microliter scale, as opposed to traditional *in vitro* platforms that operate in the milliliter scale. Therefore, the use of small volumes in BMOMs enables better recapitulation of physiologically relevant factor concentrations while avoiding sample dilutions or manipulations that may incur in sample loss (e.g., circulating tumor cells from the blood).^8^ The advantages of working with small and concentrated samples are exemplified by enzymatic processing (e.g., digestion, qPCR), where the probability of enzyme-substrate interactions increases with the solute concentration wherein the resultant reaction has improved efficiency.^9^ Furthermore, by avoiding dilution, BMOMs have also been shown to enhance paracrine signaling (i.e., soluble factor signaling between different cells) and, therefore, can facilitate studying the crosstalk between different cell types.^10,11^

Another advantage of BMOMs is their potential for scalability, which is desirable for basic studies and indispensable for extensive drug screening (e.g., Organoplate from MIMETAS).^12^ The potential of BMOMs for screening multiple therapeutic options is already being leveraged by research groups.^13,14^ However, many microfluidics-based companies are attempting to further increase the scale of multifactorial organotypic models with limited success.^15^ We anticipate that there will be further improvement in the scalability of BMOMs over time, which will require efforts in developing new materials that support mass production and operational parameters that allow use with existing high throughput instrumentation.

Additionally, we envision that microfluidics, coupled with BMOMs, could be instrumental in facilitating primary sample research. The inconsistency and labor-intensive nature of sample processing are common concerns for researchers working with primary samples. Predictable microscale physics can be leveraged to optimize laboratory procedures resulting in new strategies to standardize and automate primary sample processing (e.g., tissue mincing, enzymatic digestion, washing, and separating components of a complex mixture).^16,17^ The reproducible processing of primary tissue samples is an unmet need in the field that microfluidics could help to fill.^18^ Notably, as a response to this need, funding agencies have initiatives in the homogenization of sample processing, sample preparation, and analysis systems,^19^ and the first reports of “tissue processing devices” and sample preparation devices^20,21^ are appearing in the literature to fill this gap.

### Monitoring capabilities

Compared with *in vivo* models, BMOMs offer additional monitoring abilities. Most BMOMs are amenable to high-resolution microscopy, which can help investigate biological processes in detail (e.g., the metastatic cascade).^22,23^ Although *in vivo* models are becoming more amenable to imaging techniques (e.g., intravital imaging), they are limited by the shortcomings intrinsic to the imaging technique (e.g., breathing artifacts, limited imaging depth).^24^ This lack of real-time imaging and the complexity of the *in vivo* systems often result in the “black box” effect. This caveat is described as the user's inability to determine the relationships between input and output. Conversely, due to the increased control that the user can exert on customizing the organotypic model, the stochastic nature of BMOMs is reduced compared to *in vivo* models.

Monitoring capabilities could be further improved in BMOMs via the integration of miniaturized sensors (e.g., microfluidic flow cytometers, microfluidic Polymerase Chain Reaction devices).^25^ Some examples that illustrate the potential of sensor integration in organotypic microfluidic models are recently reported devices integrating multiple sensors (e.g., a glucose, oxygen, and pH sensor) in the same platform, which allows for fine control of the cell culture parameters in real-time.^26,27^ Other applications are CMOS-camera coupled microdevices, which are continuously monitored for higher tractability of the models and may even be automatically processed. Other monitoring examples include Raman spectroscopy integrated into microfluidic devices and miniaturized electrode-based reactive oxygen species sensors.^28^ While few of these advances have made it into mainstream laboratories, they have the potential of turning time-consuming tasks into highly standardized and automated processes, while minimizing human error and ensuring efficiency when working with small samples. However, challenges remain in the operation of these sensors (e.g., sensor biofouling)^29^ as well as in simplifying these systems to include user-friendly interfaces that can be taken advantage of in biology-focused laboratories with little need for specialized training.^30^

### A bottom-up approach to modeling

An advantage of BMOMs over *in vivo* models is their modular nature which enables scalable complexity. In BMOMs, researchers can determine the number of components desired for a specific design (e.g., a gut-liver system to study drug absorption),^31^ thereby decoupling the organs of interest to delve into basic mechanisms of disease pathology. In engineering terms, the studies enabled by BMOMs are described as bottom-up, indicating that complexity is built by adding subsequent individual physiological components.^32^ This bottom-up approach has proved useful in cell biology as it limits the system to those aspects of specific interest. For example, identifying specific cellular crosstalk between different cell types is challenging in *in vivo* models, which are top-down systems (i.e., large systems that require breaking down for study). Furthermore, BMOMs can be customized to include comprehensive and controlled environmental stimuli for mechanistic studies. Examples of environmental stimuli include mechanical cues (e.g., surface topography, stiffness, shear stress, mechanical deformations)^33–35^ and biochemical cues (e.g., pH gradients, growth factor gradients).^36–38^ Biological gradients, which are known to drive critical biological processes in tissue development;^39^ and 3D matrix architecture,^40^ which is known to drive cancer cell migration and tumor progression *in vivo*,^41^ can also be tailored through this bottom-up approach. By incorporating these cues, microfluidic organotypic models have been increasingly engineered to better resemble the microenvironment, thereby increasing their biological relevance and applicability. Studies are beginning to identify specific cell functions better mimicked in organotypic models than in traditional *in vitro* cultures. An example is a recent report that showed that cell proliferation in organotypic 3D models, which more accurately matched *in vivo* proliferation, was vastly different from the proliferation observed in traditional 2D models.^42^ Notable examples are blood and lymphatic vessel BMOMs, which have been of increasing interest in the last decade.^43,44^ Generally based on lining one or several surfaces (e.g., biocompatible materials or hydrogels) with cells of endothelial lineage (e.g., Human Umbilical Vessel Endothelial Cells or Human Lymphatic Endothelial Cells), these systems also include mechanical cues and supporting cell types^43,45,46^ The applications for these BMOMs have ranged from basic cancer biology studies to more applied drug mechanism and drug testing studies.^47–50^ More recently, tissue-specific blood vessel and lymphatic vessel BMOMs have filled an existing literature gap exploring the influence of vessel variability (e.g., lymphatic, arteries, capillaries) and tissue specificity in cancer progression, metastasis, and treatment.^40,51–55^ Finally, a recent application of BMOMs has been the mimicry of the bone microenvironment. A well-known example is the report of an organotypic microfluidic model of the bone microenvironment established by culturing bone fibroblasts in a 3D matrix.^56^ This model illustrated the bone trophism (i.e., preferential metastasis), exhibited by breast cancer cells during the metastatic cascade. More recently, microfluidic organotypic patient-specific cancer models of multiple myeloma have been developed to test treatment effectiveness *in vitro*.^56–59^

In recent years, we have witnessed a handful of studies developing patient-specific organotypic models for drug-testing applications.^43,60–63^ Although most of these studies remain at the proof of concept stage and have yet to demonstrate clinical relevance, they help to pave a path forward in how these tools can be further refined to be used clinically.

### The capacity of developing models entirely from human material

A vast majority of BMOMs report using human cell lines or patient-derived cells, which often constitutes an advantage over *in vivo* models. Despite their similarities, mice and humans present differences at many different biological levels.

First, studies have shown that the transcriptomic landscape of mice and humans is significantly different, thereby translating into physiological and functional differences.^64^ Likewise, human-to-mouse functional differences are illustrated in the immune system, where there are critical differences in both innate and adaptive immunities of humans and mice (e.g., T‐cell subsets, cytokine receptors, costimulatory molecule expression and function, Th1/Th2 differentiation, Toll‐like receptors, the NK inhibitor receptor families Ly49 and KIR).^65^ The relevance of these differences goes beyond basic studies since the immune system plays a critical role in tumor cell biology, and immunotherapy is now at the forefront of cancer treatment. An example illustrating these differences is found in the clinical trials of the anti‐CD28 monoclonal antibody TGN1412,^66^ which failed due to a cytokine storm effect that was not observed in animal models.

Second, it is known that some diseases (e.g., glioblastoma, Alzheimer's disease) cannot be modeled in mice due to the apparent mouse-to-human differences.^67^ To generate some of these models, the genome of the mice is often engineered to present mutations that have been related to the disease of interest in humans; however, the phenotypic result of these mutations does not always match the pathology observed in humans. Therefore, it remains unclear whether the relevance of the results extracted from *in vivo* models will translate to human pathophysiology.

Finally, even in those mouse models in which human cells have been implanted, the microenvironment and surrounding cells (commonly referred to as the stroma) have a mouse origin.^68^ As a consequence, animal models have limited translatability into human disease studies given the differences between humans and animals (e.g., nonconserved immune mechanisms against solid tumors). In contrast, BMOMs rely on entirely human-derived components, thereby increasing the translatability of results.

Overall, the main advantages of BMOMs are rooted in a careful balance between *in vivo* and *in vitro* models with the additional benefits derived from their small scale. Harnessing physics at the microscale in BMOMs provides many advantages: allows for a high degree of control and customizability over the building components of the model and lower reagent costs compared to traditional *in vitro* and *in vivo* models. However, BMOMs also present limitations that must be taken into consideration before future perspectives of these models can be discussed.

## LIMITATIONS OF BMOMs

Although BMOMs offer multiple advantages compared with traditional *in vitro* platforms or animal models, most of these models remain confined to engineering research and are excluded from traditional cellular and molecular biology laboratories. Adoption of these technologies has been hindered by both scientific and logistical challenges.

### Limited capacity to model multiorgan interactions and behavioral responses

Traditionally, BMOMs have focused on mimicking concrete tissue structures such as the liver sinusoid, the nephron, or the lung epithelium, which are simpler systems compared with a complete organ (e.g., liver, kidney, lung).^69^ Therefore, these models fail to fully capture the complexity of the target organ complexity and its multitude of functions (e.g., liver nutrient metabolism, drug transformation, albumin production, urea cycle).^23^ Similarly, BMOMs for human disease (e.g., alcoholic cirrhosis) commonly focus only on the affected tissue (e.g., liver), ignoring potential ramifications to other organs (e.g., alcohol abuse can lead to a severe alcohol-associated pancreatitis). Hence, identifying the required components to fully capture a specific disease state or biological function is a question that remains to be answered.^8^ Arguably, for diseases with a clear onset and defined pathophysiology, such as cystic fibrosis (CF), selectively focusing on the affected tissue might be a successful strategy. CF can be caused by mutations in the CF transmembrane conductance regulator (CFTR) gene, affecting mucus production in the lung and digestive organs, which commonly leads to life-threatening airway obstructions.^70^ Thus, BMOMs that mimic the lung epithelium physiology might suffice to study CF as well as to evaluate new therapies. Conversely, other diseases, such as cancer, have a more complex, multifactorial pathophysiology involving the interaction between multiple cell types and organs. Cancer immunotherapy might be a paradigmatic example of a complex system where the patient outcome depends on the interplay between multiple cell types (e.g., cancer, stromal, and immune cells) and biological structures (e.g., tumor vasculature, bone marrow, lymph nodes). In this scenario, BMOMs focusing on one specific tissue structure might fall short. To bridge this gap in cancer modeling, researchers have developed more sophisticated models combining multiple biological structures such as the 3D tumor tissue with the presence of blood vessel surrogates and immune cells.^23^ However, multiple steps of the immune-tumor cycle have not been yet included in the most recent models, such as antigen capture in the tumor tissue and presentation to immune cells in the lymph nodes, potential side-effects of chemo/radiotherapy in immune cell production in the bone marrow and secondary organs (e.g., thymus), or the establishment of long-term memory response (e.g., tumor vaccines).^71^

There have been some attempts to connect BMOMs mimicking different organs^72,73^ to better study these multiorgan interactions in complex disease states. To this end, two or more different tissue-specific BMOMs are combined to study the crosstalk between different organs. A representative example is the metastatic cascade, where cancer cells migrate form the primary tumor to the metastatic site. An early example explored breast cancer metastasis to bone tissue, and follow-up work included immune cell extravasation into this scenario, thereby including three different organs relevant to the metastatic cascade.^56^ More often, models have explored tumor–vascular interactions without enabling tissue-specific metastatic extravasation and colonization studies,^40,52,63,74,75^ which pales in comparison to the extensive functions observed *in vivo* (e.g., drug metabolism, nutrient processing, intravasation, cell differentiation, dissemination, and tissue-specific colonization).^76–78^

Additionally, this gap between BMOMs and human physiology becomes wider when we consider human behavior and behavioral responses. Notably, multiple diseases (e.g., irritable bowel syndrome or cancer) involve higher level functions, resulting from interactions between multiple organs and kingdoms.^79^ Notable examples include the influence of the microbiome or psychosocial stress in breast cancer prognosis and outcome.^80–82^ Some models have been developed to study simpler processes such as axon regeneration within the highly complex physiology of the brain.^83^ However, current BMOMs are yet far from successfully mimicking higher cognitive functions. In this situation, animal models might offer a more robust approach. Thus, for some applications, BMOMs still require improvement to be considered a significant alternative to animal models.

### Manufacturing and balancing required complexity with model throughput

Traditionally, soft lithography has been the most used technique to fabricate microfluidic platforms to hold BMOMs. Soft lithography relies on pouring the elastomer polydimethylsiloxane (PDMS) on top of a negative template, fabricated by UV-lithography. Next, the PDMS device is commonly bonded to a glass substrate using oxygen plasma to generate the microfluidic platform. This approach provides researchers with a relatively versatile and fast approach to prototyping. However, soft-lithography is not amenable to mass production, as compared to injection molding, one of the most common techniques used to fabricate Petri dishes and well-plates.^84^

Additionally, PDMS presents additional limitations as a material for laboratory use. PDMS is a porous material that absorbs small hydrophobic molecules, which can significantly affect cell biology. Furthermore, multiple molecules used in cell biology (e.g., Rhodamine), as well as drugs used in the clinic (e.g., doxorubicin), are absorbed into the PDMS, which will significantly decrease the effective concentration in culture.^85^ This property makes PDMS poorly suited for drug screening, and researchers have explored other alternative materials (e.g., polystyrene, PMMA)^15^ in order to overcome these limitations.

Further engineering limitations in BMOMs are illustrated in their limited throughput potential. BMOMs are a versatile tool to model complex scenarios, including interactions between multiple cell types and tissue structures (e.g., immune cells and vasculature). However, many BMOMs trade complexity for throughput potential, thereby leading to sophisticated platforms poorly suited for the parallelization and high-throughput capacity required for drug screening. Additionally, the use of specialized equipment to operate these complex models (e.g., syringe pumps, fluorescent microscope) further limits their throughput. Furthermore, most BMOMs require in-depth, hands-on training, partially explaining why they have remained limited to research environments with little translation into clinical settings. Conversely, most microfluidic platforms designed for high-throughput applications (e.g., single-cell analysis) rely on simplistic designs (e.g., microwells to capture single cells) unable to capture the complex structure and interactions of biological tissues and organs in BMOMs.^86^ Specifically, a few high throughput platforms have been developed and even commercialized to generate spheroids or luminal structures dedicated to drug testing purposes.^87–89^ These platforms present potential advantages of scale and capacity of testing multiple drug conditions and combinations, much needed for anticancer drug screening.^90,91^ Further, high throughput platforms are often conceived to be coupled with automatized pipetting systems or automatic drug gradient generation, thereby diminishing user-associated bias and error.^92,93^ However, integrating higher complexity models that include both cellular and microenvironmental cues (e.g., combination of several different cell types and structures, tubes for fluid flow, complex Extra Cellular Matrix matrices) remains a technical challenge in BMOMs. To seamlessly integrate the use of BMOMs into the biomedical community, the devices (and BMOMs) should be easy to operate and compatible with standard equipment used in research and pathology facilities.

### Future prospects

Despite the advantages of BMOMs, their implementation in research and clinical settings remains limited.

Arguably, the main barrier to a wider implementation of BMOMs is a lack of deep understanding of the critical components required for each disease model to successfully predict patient outcome. One approach that can facilitate the development of predictive BMOMs is to apply the Adverse Outcomes Pathway (AOP) developed within the field of systems toxicology. AOP defines the key events and relationships between the molecular inciting event and adverse outcome.^94^ AOP is an approach to drug testing that integrates a comprehensive understanding of the mechanism of action of the drug. The application of the AOP framework has been suggested for the development of patient-specific BMOMs. The modular and bottom-up nature of BMOMs enables the integration of the main tissues or biological components playing a role in the targeted biological process.^95^ AOP can guide the development of BMOMs to balance complexity, throughput, and straightforward operation in a more standardized manner across different research groups. This bottom-up, reverse engineering approach may be laborious but is likely required for the rational design of BMOMs, which will result in long-term cost-saving, facilitate commercialization, and eventually result in implementation in clinical settings.

**FIG. 2.**
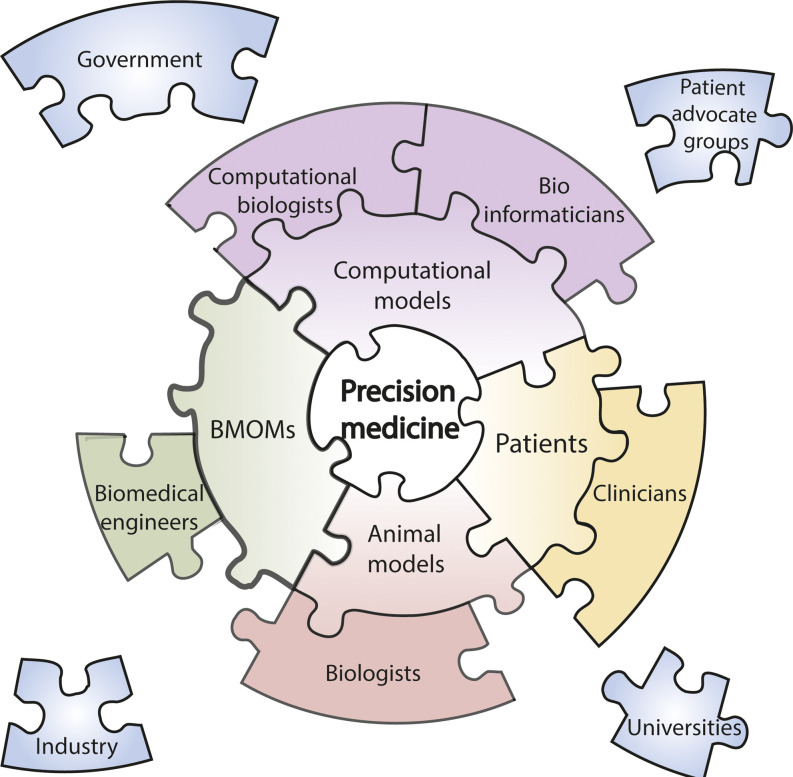
The precision medicine “puzzle.” We envision that precision medicine will rely on the convergence of four main pillars: patients, animal models, organotypic microscale models, and computational models. Interdisciplinary and collaborative work will be necessary to achieve this goal. Further, active collaboration of universities, industry, patient advocate groups and the government remain to be pieced into this concerted effort.

The complex process of rational design for BMOMs may benefit from the integration of computational systems biology, which aims to mathematically model complex, nonlinear biological systems.^96^ The integration of computational systems biology and BMOM development will likely require continuous and close collaboration between computational and brick-and-mortar biology researchers to ensure accurate *in silico* modeling of BMOMs. For example, studies are being continued to illustrate the importance of the microbiome and cross-kingdom interactions, as well as stress, diet, and environment^80^ in understanding human diseases. These newly found interactions may need to be included and evaluated both in *in silico* and “wet” models to unravel their importance in a specific mechanism according to AOP. Thus, it is unlikely that we will be able to develop the ideal BMOM without the help of animal models and clinical studies. A synergy between the engineering community, biologists, computational biologists, and clinicians can allow the information obtained from human and animal studies to be incorporated into BMOMs and computational models and vice versa. Furthermore, developing predictive patient-specific BMOMs to achieve the goals of precision medicine poses even greater challenges that can only be overcome through a concerted and multidisciplinary effort in research, engineering, industry, and patient advocate foundations (Fig. 2).^97–101^

## AUTHORS' CONTRIBUTIONS

J.M.A., K.Y.P., and M.V. contributed equally to this work.

## Data Availability

Data sharing is not applicable to this article as no new data were created or analyzed in this study.
